# Development of a tablet PC-based portable device for colorimetric determination of assays including COVID-19 and other pathogenic microorganisms

**DOI:** 10.1039/d0ra05866a

**Published:** 2020-09-04

**Authors:** Woo Sik Yoo, Hyung Soo Han, Jung Gon Kim, Kitaek Kang, Hyo-Sung Jeon, Jin-Young Moon, Hyeonmi Park

**Affiliations:** WaferMasters, Inc. Dublin CA 94568 USA woosik.yoo@wafermasters.com; Department of Physiology, Clinical Omics Institute, School of Medicine, Kyungpook National University Daegu Republic of Korea; M Monitor, Inc. Daegu 47213 Republic of Korea

## Abstract

There has been a strong and urgent demand to diagnose community transmission-driven coronavirus disease 2019 (COVID-19) after it crossed borders. A large number of rapid and accurate tests and diagnoses are required at drive-through test stations, community clinics and hospitals. Isothermal amplification technology, such as loop-mediated isothermal amplification (LAMP) and recombinase polymerase amplification (RPA), provides excellent alternatives for resource limited test environments. LAMP has been shown to be comparable with polymerase chain reaction (PCR) and can be performed in less than 30 min by non-laboratory staff without ribonucleic acid (RNA) extractions commonly associated with PCR. LAMP tests on assays with SARS-CoV-2 and other pathogenic microorganisms, such as Dengue, Malaria, and Influenza viruses and *Helicobacter pylori* show color changes allowing test results to be interpreted by the color change of the assays. However, visual inspection of a large number of assays is prone to human error and manual record keeping makes test result tracking for an epidemiologic investigation very difficult and inefficient. The epidemiologic investigation is an essential part of the fight against community transmission-driven viruses. We have developed a very accurate and reliable, human error free, tablet PC-based portable device for colorimetric determination of assays including SARS-CoV-2 and other pathogenic microorganisms.

## Abstract 2

There has been a strong and urgent demand to diagnose community transmission-driven coronavirus disease 2019 (COVID-19) after it crossed borders. Tablet PC-based portable device for colorimetric determination and 3D printed sample holders.

## Graphical abstract

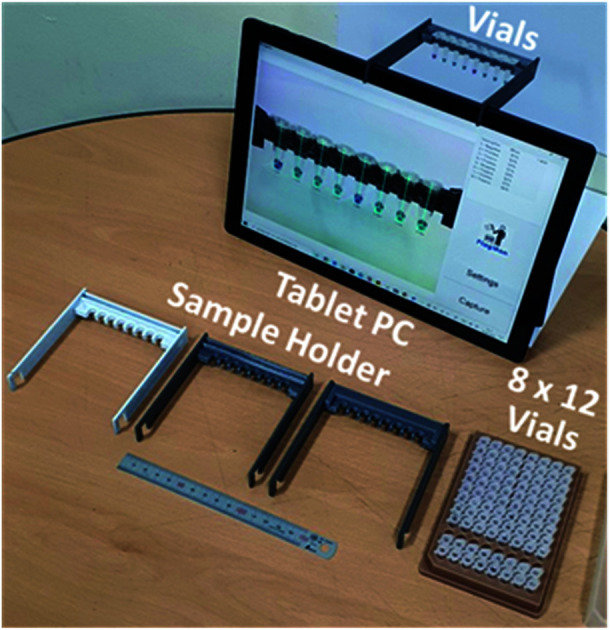

## Introduction

A novel coronavirus (SARS-CoV-2) originated from an epicentre in Wuhan province in China. Due to its rapid spread across borders, it has pushed the capacity of the global public health community to its sheer limits and led to bans on travel and public gatherings. It also has negatively impacted the world economy.^1^ On January 30, 2020, the World Health Organization (WHO) declared COVID-19 as a global public health emergency.^2^ COVID-19 is characterized with high morbidity and low mortality therefore representing a great threat, particularly to immunocompromised, elderly people, and individuals with pre-existing health problems. As of June 12, 2020, there were over 7.7 million confirmed cases of COVID-19 and ∼430 000 deaths recorded worldwide.^3^ Approximately 4.0 million patients have recovered. New cases are approaching 150 000 per day and approximately 5000 daily deaths are reported. As of August 24, 2020, 23.7 million confirmed cases and ∼814 000 deaths have been reported. COVID-19 is still spreading very rapidly worldwide.^3^

The spread of infections at a church in a Korean city, Daegu, drove a massive spike in cases in Korea beginning in February 2020. The outbreak initially pushed the tally of confirmed cases much higher than anywhere else outside of China, before the country used widespread testing and social distancing measures to bring the numbers down. It once had the biggest coronavirus outbreak outside of China. Now it has reported zero new cases.^4,5^

Daegu is a city in Korea where two of three co-authors are located. The city was host to the first large coronavirus outbreak outside of China. The city has reported a total of 6807 cases since the outbreak started there in late February. On April 10, 2020, it reported zero new cases. Drive-through (DT) screening centres for COVID-19 were introduced on February 23, 2020 at Kyungpook National University Chilgok Hospital, Daegu, Korea, where the huge COVID-19 outbreak occurred, as a safe and efficient screening system against a massive community outbreak.^6^ On April 10, 2020 zero new cases were reported for the first time since February, as new infections across the country dropped to record lows. Having diagnostic capacity at scale is key to epidemic control and contact tracing is also very influential to maintain epidemic control, as is case isolation. Widespread testing and social distancing measures are credited for the success in curtailing the spread of the virus.^6^

For stopping the spread of COVID-19, the availability of reliable and aggressive testing is imperative. Samples collected from suspected cases are presented at well-equipped community clinics and hospitals. They are processed at a centralized clinical laboratory, where expensive PCR equipment and technical expertise are available. Typical turn-around-time for the test results is up to 72 hours.^1^ Such a long waiting time for testing and a long turn-around-time for the test results could lead to anxiety and continued spread of the virus. Assuring self-isolation after the standard real-time RT-PCR at a centralized clinical laboratory requires additional resources and is difficult to impose. An appropriate point of care COVID-19 test can help reduce anxiety and eliminate prolonged turn-around time. It will greatly help reduce the chance of spreading the virus. A rapid, robust, and cost-effective point of care device can be used onsite and in the field. Highly trained personnel to operate sophisticated testing equipment are not required. Having an appropriate point of care device is crucial and urgently needed for the fast detection of COVID-19.^1,4–8^

As the growing number of suspected COVID-19 cases exceeds the capacity of many hospitals, many patients remain untested, impeding efforts to control the disease. A rapid, point-of-care (POC) diagnostic for the COVID-19, which we will propose, using a loop-mediated isothermal amplification (LAMP) method of detection can be of great use.^9–12^ The LAMP test can be done in less than half an hour.^7–12^ Test results can often be interpreted by colour change using the naked eye. However, for approximately 5 percent of tests, it may be difficult to judge the results with an unaided eye due to colour ambiguity and turbidity.

In this study, we have developed a very accurate and reliable, human-error free, tablet PC-based portable device for colorimetric determination of assays including COVID-19 and other pathogenic microorganisms for combination with a POC diagnostic device that can be used in various medical occasions including resource-limited settings and remote areas. It can be fast, adaptable and sensitive for the detection of a wide range of different assays in a very cost-effective way. It will also generate test results and a summary with test images, daily activity log, statistical analyses and customized reports.

## Colorimeter design considerations

To enable prototyping a POC colorimetric device, a battery-powered, wirelessly connected and ready to use computer with an integrated camera(s) such as a smart phone, notebook personal computer (PC) and/or tablet PC are excellent candidates from the computing and communication capability point of view.^13–16^ Accuracy and repeatability of colorimetric colour sensing is strongly dependent on ambient light conditions and light source configurations relative to the assays in vials (cuvettes or test tubes) and camera specifications. A stable and reliable light source including natural lighting and/or indoor lighting is strongly desired. To reduce interference from ambient light and make colour sensing stable and reliable, the distance between sample(s) and camera must be fixed.^15^ These requirements make the use of smart phones for the POC applications less desirable. Notebook PCs come with an integrated camera on the display side, thus an external camera is required. Some tablet PC models have two integrated cameras (one on the front side and one on the backside (display side)). Thus, the tablet PC-based colorimeter design is a natural choice for a simple and reliable engineering solution. A sample holder which keeps the samples at a fixed distance and consistent ambient light conditions need to be fabricated and placed in front of the front side integrated camera.

**Fig. 1:**
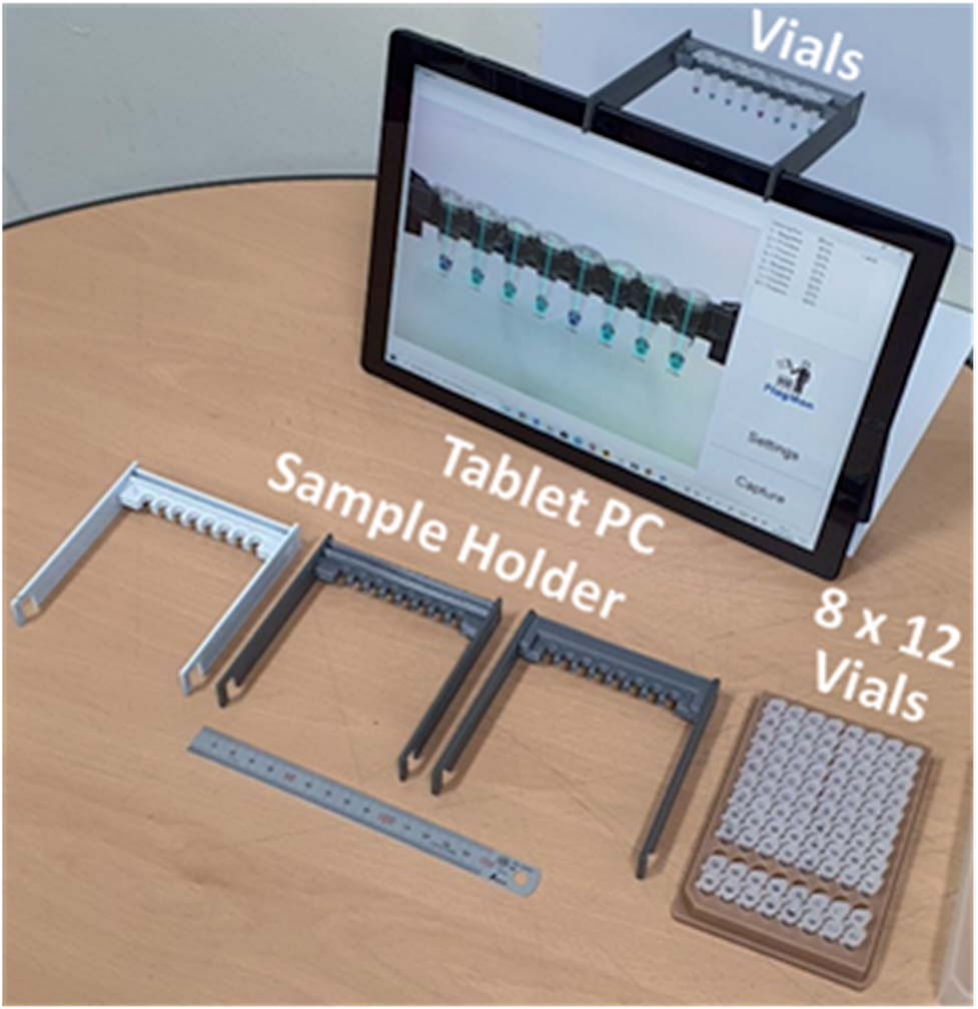
Tablet PC-based portable device for colorimetric determination and sample holders fabricated using 3D printing technology.

The sample holder for the tablet PC-based colorimetric device can be fabricated using three-dimensional (3D) printing technology. A tablet PC-based portable device (FlagMan) for colorimetric determination and sample holders fabricated using 3D printing technology are shown in Fig. 1. Sample holder design can be modified based on the application needs (for example, dimensions of vials, number of test vials per measurement and so on). The sample holders in Fig. 1 hold eight vials and are placed in front of the front side integrated camera. A set of eight vials can be tested simultaneously. Manual loading and unloading of eight vials can determine ‘positive’ and ‘negative’ test results. If the colour of vials does not belong to the judgment criteria, judgment results are displayed as ‘unknown’.

Automatic image capture of eight vials with colorimetric determination results and diagnosis summary per image and a daily diagnosis summary (for positive, negative, unknown or all results) are reported with colorimetric measurement details for tracing and verification of results.

## Experiment

### Assay preparation and general description of experiment

**Fig. 2:**
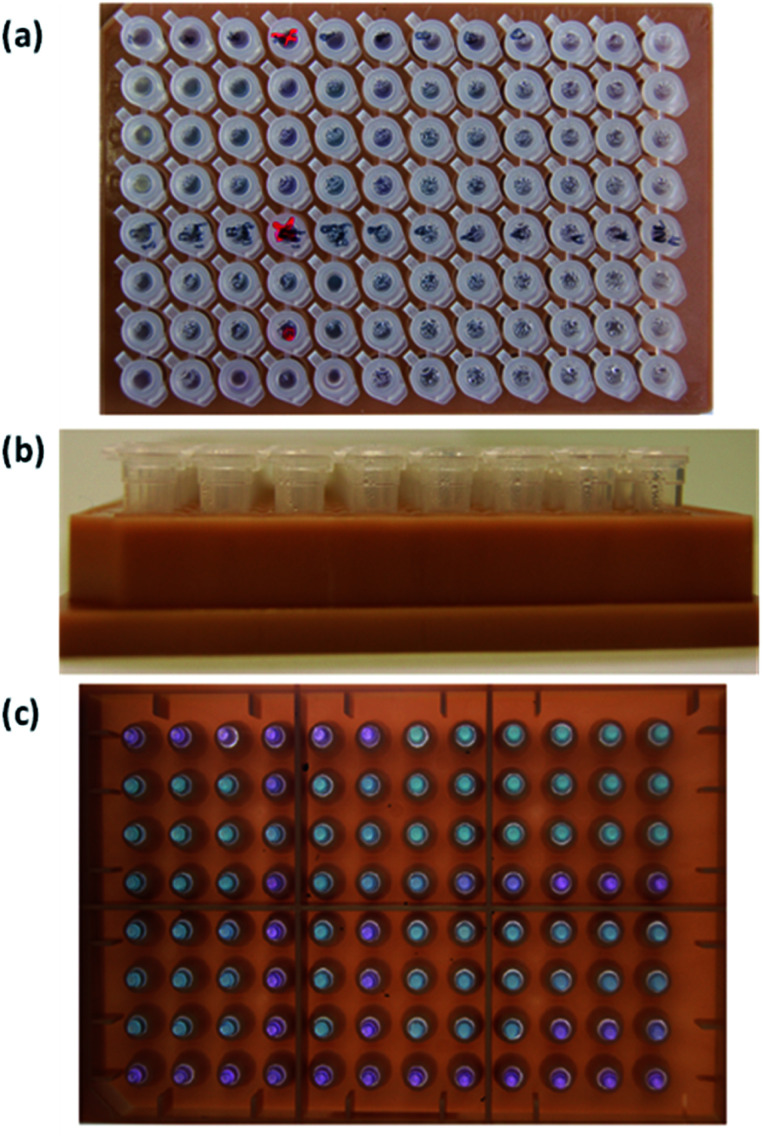
Photographs of 8 × 12 assays used in this study. (a) Top view, (b) side view and (c) bottom view.

Assays for COVID-19 (Mmonitor's Isopollo® COVID-19 detection kit (premix))^17^ were used for colorimetric detection. Fig. 2 shows an 8 × 12 vial kit for 96 POC tests with negative and positive reactions. Top view, side view and bottom view of the 8 × 19 vial kit are seen in Fig. 2(c); note there are variations in colour. The COVID-19 test kit can check for infection through colour change. Negative result shows purple colour and positive result shows sky blue. The result can be interpreted by the naked eye when colour change is obvious. However, subtle and ambiguous colour makes interpretation very difficult and can cause misinterpretation by human error.

Since the colour change of assays originate from the synthesis of larger amounts of DNA and a visible by-product, the degree of colour change from purple (negative) to sky blue (positive) is dependent on the density of by-product. Goto *et al.*^12^ have reported colorimetric detection of LAMP reactions by using hydroxy naphthol blue. In the report, a positive reaction is indicated by a colour change from ‘violet’ to ‘sky blue’. The same colour for negative results is referred to as ‘purple’ or violet’ from different research groups. This ambiguity of verbal descriptions of colour can cause human errors in interpreting results during hectic times at POC sites under emergency situations during a pandemic. Quantitative characterization of colour in assays is very important to reduce human errors and provide statistical analysis for database collection.

### Collection and preparation of samples

RNA extract samples were provided from Kyungpook National University Hospital (KNUH). After collection of swap sample, RNA was isolated using QIAamp Viral RNA Mini Kit (52906, Qiagen) and stored at −80 °C until experiment. Every experimental procedure was approved by Ethics Committee and Institutional Review Board of KNUH (IRB number: KNUH 2020-03-003). All experiments were carried out in accordance with relevant guidelines and regulations.

### Assay using Isopollo® COVID-19 detection kit (premix)

Isopollo® COVID-19 detection kit (premix) is an *in vitro* diagnostic kit for qualitative analysis to diagnose severe acute respiratory syndrome coronavirus 2 (SARS-CoV-2) infection of clinical specimens from human nasopharyngeal and oropharyngeal swab, sputum, and bronchoalveolar lavage by reverse transcription loop-mediated isothermal amplification (RT-LAMP) and it has high specificity for targeted RNA because 6 primer sets selectively detect RdRP and N genes of SARS-CoV-2. After completion of the reaction, the results can be confirmed immediately by colour change. The negative reaction shows purple colour while positive reaction shows blue. All the assay was performed following manufacturer's manual. For the most of the assay EDX SARS-CoV-2 Standard (Exact Diagnostics)^18^ was used as a template of the RT-LAMP. EDX SARS-CoV-2 Standard is manufactured with synthetic RNA transcripts containing five gene targets (E, N, ORF1ab, RdRP and S Genes of SARS-CoV-2). RNA concentration used for the assay was set at 40 copies per μl, 20 copies per μl, 10 copies per μl, 5 copies per μl and 0 copies per μl. The limitation of detection of the kit was between 10 and 5 copies per μl. For some experiment clinical samples were tested using both Isopollo® COVID-19 detection kit (premix) and Standard real time PCR assay approved by Korean FDA (Food and Drug Administration) to compare the results.

### Factors of colorimetric characteristics

Main colour, balance of primary colours (RGB: red, green and blue) of light, and the degree of transparency or turbidity of photographic images of assays from an integrated camera of the tablet PC-based portable device are collected and analysed for quantitative colorimetric determination. The colour of the captured images can be expressed into hue-saturation-value (HSV) coordinates.^15^ However, the HSV coordinates use different ranges for each parameter and are inconvenient for comparing colorimetric characteristics. Hue is expressed in the range of 0–360°, while saturation and value (lightness or brightness) are expressed in 0–100%.

To make the colour comparison easier, we have introduced new categories of DEF (decisive, effective and fuzzy) colour determination factors in the range of 0–100. The factors DEF are somewhat similar to HSV. The factor *D* primarily depends on main colour (*i.e.* wavelength of main colour peak). The factor *E* expresses the degree of asymmetry in RGB intensity of assays, and the factor *F* is related to the degree of clarity. Purple or violet (negative) will have large *D* values and sky blue (positive) will have small *D* values. Pure colour or colour with narrow band will have large *E* values and mixed colour will have small *E* values. Clear assays will have large *F* values and opaque assays will have small *F* values. The main colour peak (*D* value), purity of colour (*E* value) and clarity (*F* value) can be used together for colorimetric determination of assays.

### Collection of colorimetric database for assays

**Fig. 3:**
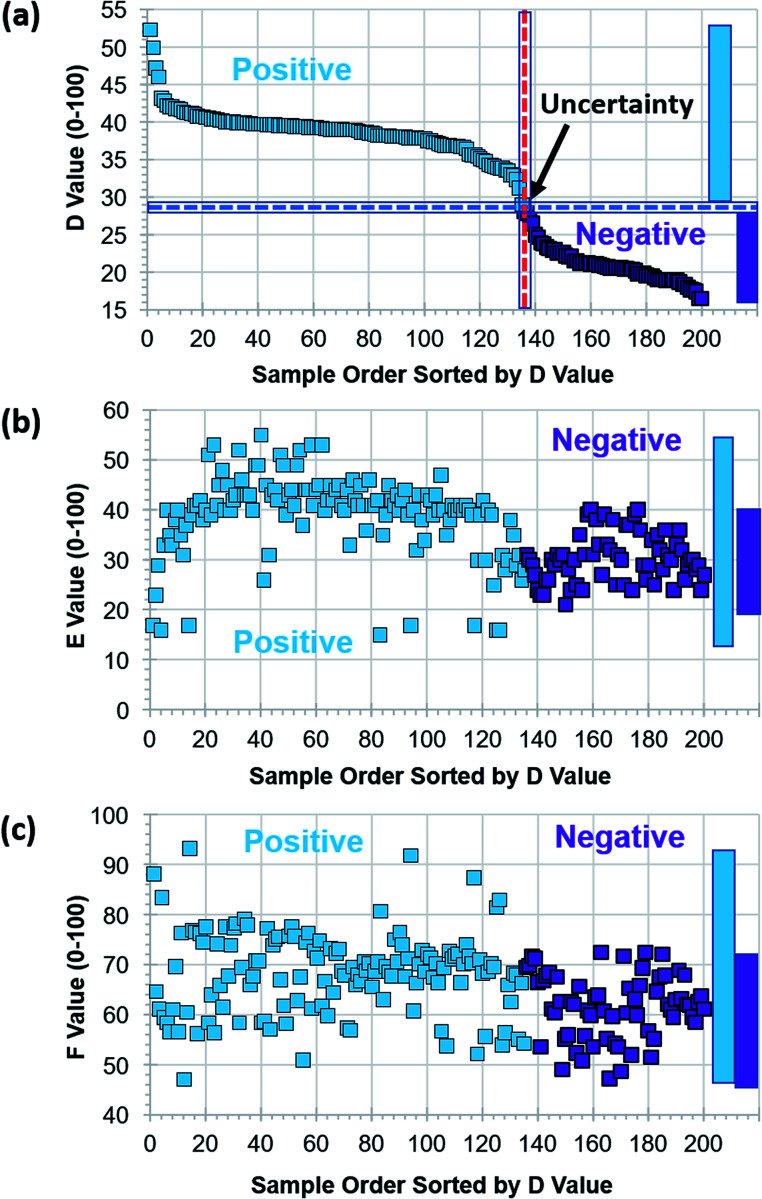
Colorimetric characterization summary of 200 assays with negative and positive reactions. Samples are sorted by *D* value to demonstrate primary factor and windows for test result judgment. (a) Range of *D* values (28.6 ± 0.5) is indicated as uncertainty zone. Ranges of (b) *E* values and (c) *F* values for positive and negative reactions overlap.

Six 8 × 12 assay kits, 576 vials, with positive and negative reactions were photographed using the tablet PC-based portable colorimetric device (Fig. 1) and their colorimetric characteristics analysed in the proposed DEF coordinates. Variations in main colour peak (*D* value), purity of colour (*E* value) and clarity (*F* value) of negative and positive assays are characterized. Once the colorimetric database is collected, test result judgment criteria and windows for *D*, *E*, *F* values can be identified. Since the colour change from purple to sky blue is the main judgment criteria for LAMP assays, the values for factors *D*, *E*, and *F* of the first 200 assays are sorted by *D* value (Fig. 3).

The *D* value for negative assays was in the range of 16.5–28.1 and the *D* value for positive assays was in the range of 29.1–52.4. As the centre wavelength of the main colour peak increases from purple to sky blue, the *D* value also increases. The *D* value boundary for negative and positive results was around 28.6 and there was no overlap (see the vertical axis of Fig. 3(a)). The test results can be primarily determined by the *D* value (main colour peak) from the colorimetric analysis data of assays. Although the *E* (purity of colour) and *F* (clarity) values also showed the difference between negative and positive test results, windows for negative and positive results are overlapped. Since the purity and clarity (or turbidity) of assay colour also indicates the degree of reaction, all three factors of *D*, *E* and *F* should be utilized not only for the negative/positive determination but also for characterizing details of reaction. Although there is no overlap, the test results with the *D* values of 28.6 ± 0.5 (range of 1.0 in the *D* value) must to be examined carefully, and perhaps, retested.

## Results and discussions

### Colorimetric diagnosis tool calibrations

**Fig. 4:**
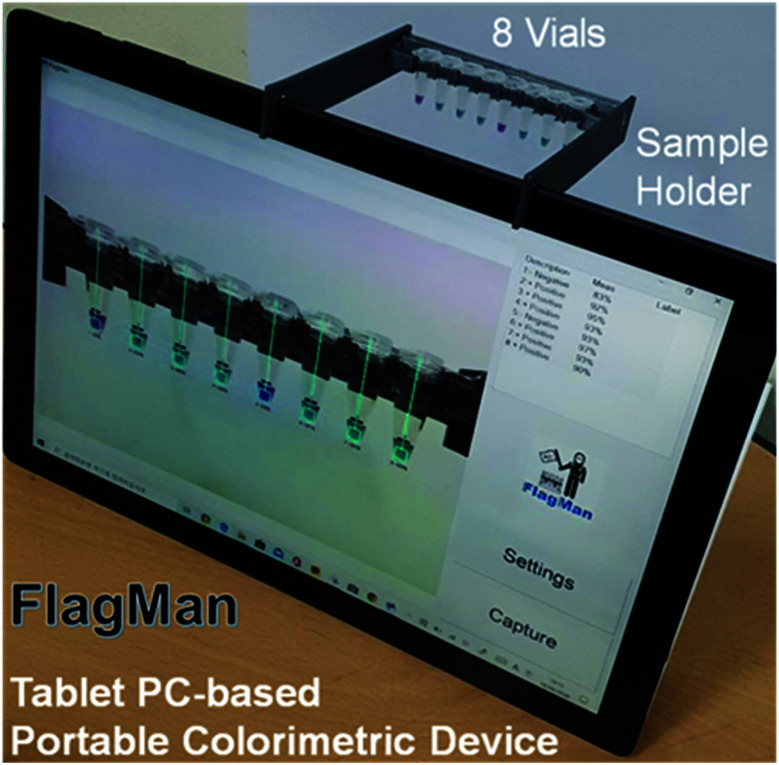
Tablet PC-based portable colorimetric detection device with eight colorimetric assays.

Based on the calorimetric database for test result determination criteria, test algorithms were written for multiple tablet PC-based portable devices (Fig. 4). The devices have been tested for three months in three different locations under various lighting conditions. Natural lighting, indoor fluorescent lighting, light emitting diode (LED) lighting and light box lighting conditions were tested. There were slight variations in *E* (purity of colour) and *F* (clarity) values depending on lighting configurations and conditions. However, the influence on the most important factor of *D* value for the main colour peak was very limited and test result interpretations under tested lighting configurations and conditions were not affected. After lengthy evaluation of the device performance, we have optimized test result interpretation algorithms for accuracy, repeatability and stability improvement. Initial worries about using a smart phone-based portable device without using lighting environment-controlled compartments was not a factor for the tablet PC-based device with an appropriate sample holder. Design and implementation of customized vial holders for the tablet PC-based device greatly stabilized the test results and made it POC application worthy.

### Automatic report generation

**Fig. 5:**
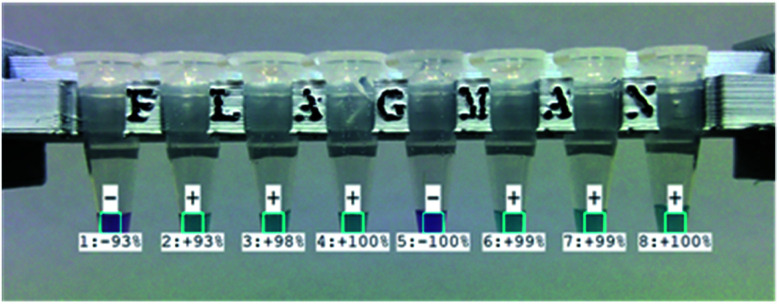
Automatically captured and exported image of eight vials with test results and levels of confidence.

To make the device more user friendly, automatic image capture and analysis functions were implemented. Images of vials with test results are automatically exported as image files for record keeping and test history tracing (Fig. 5). The device can read barcode ID (identifier) information on the vials. A summary of daily test result details in test IDs, RGB values, DEF values, and test results with test confidence are automatically exported in four different CSV (comma separated value) files of positive, negative, unknown and all test results. The exported CSV files can be opened by spreadsheet applications for statistical analysis and tracing.

### Measurement consistency and shelf life

To investigate the effect of lighting conditions and the effect of time to test after vial preparation, 260 vials with positive and negative results were tested under various lighting conditions over a 15 day period. Due to the built-in auto contrast, white balance and exposure functions of the tablet PC, colorimetric test results were almost identical under natural lighting, fluorescent lighting and white LED (light emitting diode) lighting conditions. As long as the broad light spectrum is in the visible wavelength region (400–700 nm), colorimetric test results were consistent within ±0.5 in the *D* value.

**Fig. 6:**
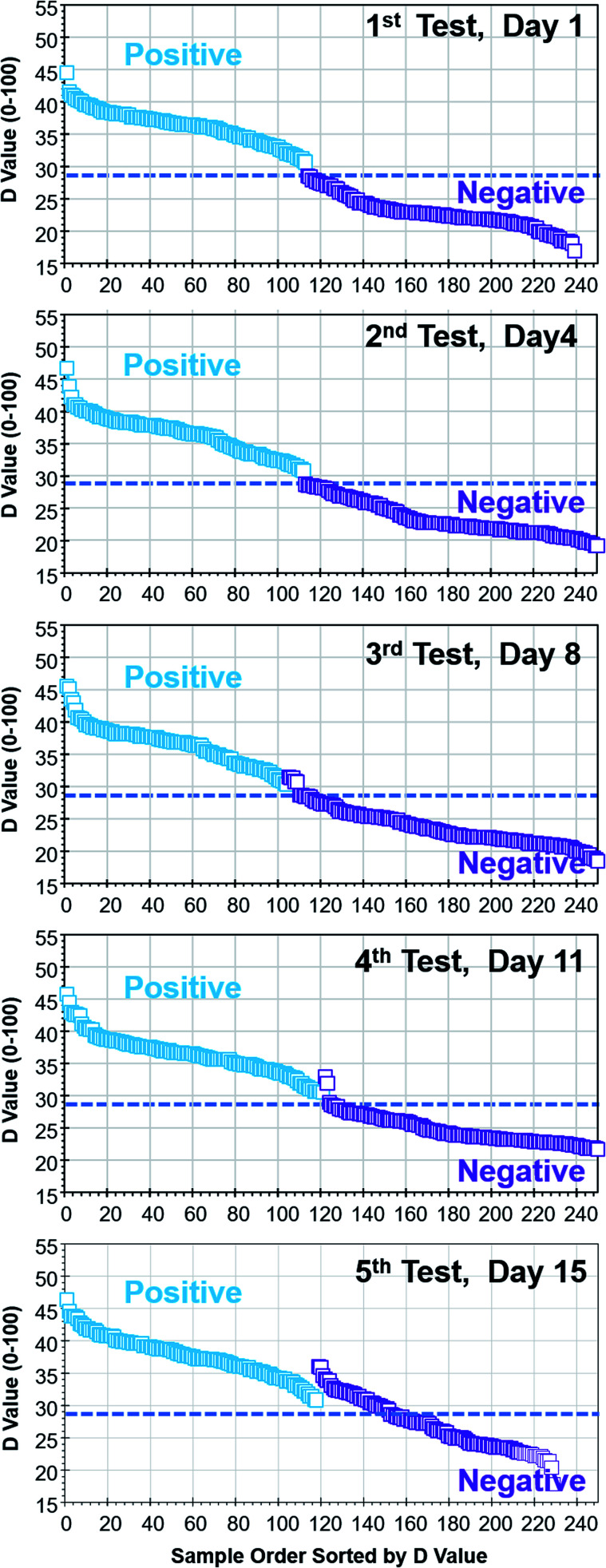
Effect of sample aging on colour change and test results (same set of vials were tested five times in a 15 day period).

It is known that the colour of assays goes through slight colour change with time due to the oxidation. Fig. 6 summarizes the five sets of tests over a 15 day period under natural lighting through a lab window. The horizontal lines were added at the *D* value of 28.6, which is the test results judgement threshold value. The colorimetric test results were consistent until the 2nd set of tests on the 4th day. If samples were tested within four days, colour change is minimal and consistent test results can be obtained. The colour change of assays due to oxidation was observed from the 3rd set of tests on the 8th day and could result in misjudgement of test results on a few vials near the judgement threshold of the *D* value (28.6 ± 0.5). It should be noted that the number of valid samples differ from test to test due to the evaporation of assays.

**Fig. 7:**
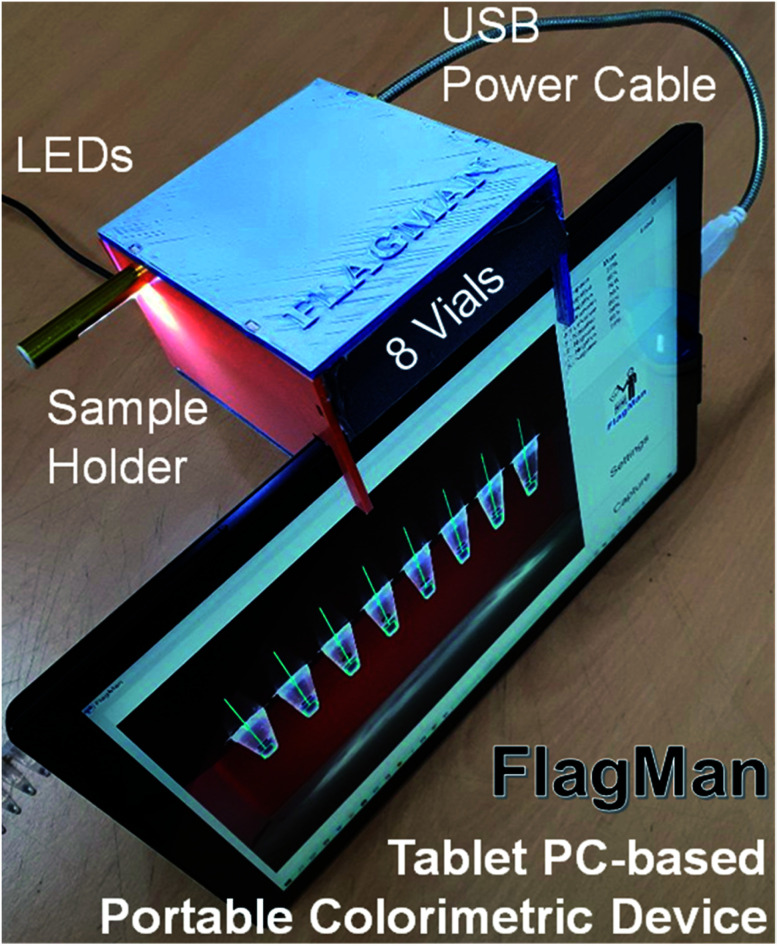
Tablet PC-based portable colorimetric detection device with an enclosed sample holder designed for consistent lighting conditions.

For more consistent results, under identical lighting conditions, a test box with a light source and lid (Fig. 7) can be attached to the tablet PC instead of the open space sample holders shown in Fig. 1. It is a matter of choice depending on the medical care environment. For remote and isolated medical care centres, electricity supply, even for the light source, can also be a problem.

### Future applications

Besides LAMP tests on assays with COVID-19, colour change due to the other pathogenic microorganisms, such as Dengue, Malaria, and Influenza viruses and *Helicobacter pylori* can be used for detection of infection. Multiple viruses can also be simultaneously detected using an appropriate number of vials. Vial holders can be modified and fabricated by 3D printing technology as needed. Test result judgment criteria can also be modified or optimized. Certification of the tablet PC-based colorimetric detection device as a clinical device with software for medical applications is in progress. This battery operated and natural lighting operated portable device will greatly help medical professionals in all occasions, including temporary DT test stations and remote locations with limited access of electricity. Automatic test image record keeping and test result summary reporting functions will reduce human error and help tracing test records. It is very promising technology for POC applications. The flexibility of customization is also a very attractive feature of this approach.

## Conclusions

There has been a strong and urgent demand to diagnose community transmission-driven COVID-19. A large number of rapid and accurate tests and diagnoses are required at drive-through test stations, community clinics and hospitals around the world. The epidemiologic investigation and isolation of cases are essential parts of fighting against community transmission-driven viruses.

LAMP, which is comparable with polymerase chain reaction (PCR), can be performed by checking colour change in less than 30 min by a non-laboratory staff. Visual inspection of a large number of assays is prone to human error and manual record keeping also adds to staff workloads. To reduce human error and workload, we have developed and successfully demonstrated a very accurate and reliable, tablet PC-based portable device (FlagMan) for colorimetric determination of assays including COVID-19 and other pathogenic microorganisms. The tablet PC-based colorimetric detection device is very promising for many applications in the medical field.

## Conflicts of interest

There are no conflicts to declare.

## Supplementary Material
